# Antibacterial, antifungal and *in vitro* antileukaemia activity of metal complexes with thiosemicarbazones

**DOI:** 10.1111/jcmm.12508

**Published:** 2015-02-24

**Authors:** Elena Pahontu, Felicia Julea, Tudor Rosu, Victor Purcarea, Yurie Chumakov, Petru Petrenco, Aurelian Gulea

**Affiliations:** a Inorganic Chemistry Department, Faculty of Pharmacy, University of Medicine and Pharmacy “Carol Davila”Bucharest, Romania; b Coordination Chemistry Department, Moldova State UniversityChisinau, Republic of Moldova; c Inorganic Chemistry Department, Faculty of Chemistry, University of BucharestBucharest, Romania; d Complementary of Sciences Department, Faculty of Medicine, University of Medicine and Pharmacy “Carol Davila”Bucharest, Romania; e Institute of Applied Physics, Academy of Sciences of MoldovaChisinau, Republic of Moldova

**Keywords:** thiosemicarbazone, crystal structure, Cu(II), V(V) and Ni(II) complexes, antimicrobial activity, antiproliferative activity

## Abstract

1-phenyl-3-methyl-4-benzoyl-5-pyrazolone 4-ethyl-thiosemicarbazone (HL) and its copper(II), vanadium(V) and nickel(II) complexes: [Cu(L)(Cl)]·C_2_H_5_OH·(**1**), [Cu(L)_2_]·H_2_O (**2**), [Cu(L)(Br)]·H_2_O·CH_3_OH (**3**), [Cu(L)(NO_3_)]·2C_2_H_5_OH (**4**), [VO_2_(L)]·2H_2_O (**5**), [Ni(L)_2_]·H_2_O (**6**), were synthesized and characterized. The ligand has been characterized by elemental analyses, IR, ^1^H NMR and ^13^C NMR spectroscopy. The tridentate nature of the ligand is evident from the IR spectra. The copper(II), vanadium(V) and nickel(II) complexes have been characterized by different physico-chemical techniques such as molar conductivity, magnetic susceptibility measurements and electronic, infrared and electron paramagnetic resonance spectral studies. The structures of the ligand and its copper(II) (**2**, **4**), and vanadium(V) (**5**) complexes have been determined by single-crystal X-ray diffraction. The composition of the coordination polyhedron of the central atom in **2**, **4** and **5** is different. *The tetrahedral coordination* geometry of Cu was found in complex **2** while in complex **4**, it is square planar, in complex **5** the coordination polyhedron of the central ion is distorted square pyramid. The *in vitro* antibacterial activity of the complexes against *Escherichia coli*, *Salmonella abony*, *Staphylococcus aureus*, *Bacillus cereus* and the antifungal activity against *Candida albicans* strains was higher for the metal complexes than for free ligand. The effect of the free ligand and its metal complexes on the proliferation of HL-60 cells was tested.

## Introduction

The chemistry of the transition metal complexes of thiosemicarbazones became largely appealing because of their broad profile of pharmacological activity that provides a diverse variety of compounds with different activities 1–4. Some of the detected biological activities of the thiosemicarbazones and their complexes with transition metal ions are antibacterial, antifungal, antiarthritic, antimalarial, antitumor, antiviral and anti-HIV activities 5–10.

Thiosemicarbazone derivatives containing a 4-acyl-2-pyrazolin-5-one moiety form an important class of organic compounds because of their structural chemistry and biological activities 11. In the field of anticancer research, the pyrazolones exhibited promising antiproliferative activity against human myelogenous leukaemia HL-60 12. The co-ordinating property of the 4-amino-2,3-dimethyl-1-phenyl-3-pyrazolin-5-one ligand has been modified to give a flexible ligand system, formed by condensation with a variety of reagents such as aldehydes, ketones 13–15, thiosemicarbazides and carbazides, *etc*. 16–18. The biological properties of thiosemicarbazones are often related and modulated by metal ion coordination.

This work is the result of our systematic studies in this field 19–21. In this study, we report the synthesis, spectral studies and crystal structures of Cu(II), V(V) and Ni(II) complexes with 1-phenyl-3-methyl-4-benzoyl-5-pyrazolone 4-ethyl-thiosemicarbazone.

## Materials and methods

### Antibacterial and antifungal activity

The free ligand and metal complexes synthesized were tested for their *in vitro* antibacterial activity against Escherichia coli (O-111), Salmonella abony, Staphylococcus aureus (209-P), Bacillus cereus and their anti-fungal activity against Candida albicans strains using the paper disc diffusion method 22 (for qualitative determination) and with serial dilutions in liquid broth 23 [for the determination of minimum inhibitory concentration (MIC) and minimum bactericidal concentration (MBC)]. Furaciline and nistatine were used as reference substances.

Qualitative determination of antimicrobial activity was carried out using the disk diffusion method. Suspensions in sterile peptone water from 24 hrs cultures of microorganisms were adjusted to 0.5 McFarland. Mueller-Hinton Petri dishes of 90 mm were inoculated using these suspensions. Paper disks (6 mm in diameter) containing 10 ml of the substance to be tested (at a concentration of 2048 mg/ml in dimethylsulfoxide) were placed in a circular pattern on each inoculated plate. The plates were incubated at 37°C for 24 hrs. The results were read by measuring the diameters of the inhibition zones generated by the tested substances, using a ruler. Determination of MIC (mg/ml) was carried out using serial dilutions in liquid broth method. The materials used were 96-well plates, suspensions of microorganism (0.5 McFarland), Mueller-Hinton broth (Merck, Bucharest, Romania) and solutions of the substances to be tested (2048 mg/ml in dimethylsulfoxide). The following concentrations of the substances to be tested were placed in the 96-well plates: 1024; 512; 256; 128; 64; 32; 16; 8; 4; 2 mg/ml. After incubation at 37°C for 18–24 hrs, the MIC for each tested substance was determined by macroscopic observation of microbial growth, which corresponded well with the lowest concentration of the tested substance where microbial growth was clearly inhibited.

The antimycotic properties were determined in liquid Sabouroud medium (pH 6.8). The inoculates were prepared from fungal stems which were harvested from 3 to 7 day-old cultures. Their concentration in suspension was (2–4)·10^6^ colony forming units per millilitre. Sowings for fungi and micelles were incubated at 37°C during 7 and 14 days, respectively.

### Antiproliferative activity

#### Cell culture

Human promyelocytic leukaemia cells HL-60 (ATCC, Rockville, MD, USA) were routinely grown in suspension in 90% RPMI-1640 (Sigma-Aldrich, Saint Louis, MO, USA) containing *L*-glutamine (2 nM), antibiotics (100 IU penicillin/ml, 100 μg streptomycin/ml) and supplemented with 10% (v/v) foetal bovine serum, in a 5% CO_2_ humidified atmosphere at 37°C. Cells were currently maintained in continuous exponential growth with dilution of the cells in culture medium twice a week.

#### Cell proliferation assay

The cell proliferation assay was performed with 3-(4,5-dimethylthiazol-2-yl)-5-(3-carboxymethoxyphenyl)2-(4-sulfophenyl)-2H-tetrazolium (MTS) (Cell Titer 96 Aqueous; Promega, Madison, USA USA), which allowed us to measure the number of viable cells. In brief, triplicate cultures of 1 × 10^4^ cells in a total of 100 μl medium in 96-well microtiter plates (Becton Dickinson and Company, Lincoln Park, NJ, USA) were incubated at 37°C, in 5% CO_2_. Compounds were dissolved in DMSO to prepare the stock solution of 1 × 10^−2^ M. These compounds were diluted to the appropriate concentration (1 or 10 μM) with culture media, added to each well and incubated for 3 days. Following each treatment, 20 μl MTS was added to each well and incubated for 4 hrs. MTS is converted to water-soluble coloured formazan by dehydrogenase enzymes present in metabolically active cells. Subsequently, the plates were read at 490 nm using a microplate reader (Molecular Devices, Sunnyvale, CA, USA). The results were reported as the percentage of cell proliferation inhibition compared to the control (basal cell proliferation = 100%).

#### Chemistry

The substances 4-ethyl-thiosemicarbazone (Sigma-Aldrich) and 1-phenyl-3-methyl-4-benzoyl-5-pyrazolone (Merck) were used as received. The metal salts CuCl_2_·2H_2_O, CuBr_2_, Cu(OAc)_2_·H_2_O, Cu(NO_3_)_2_·3H_2_O, (VO)SO_4_·2H_2_O, NiCl_2_·6H_2_O (Merck) were used as supplied. Solvents used for the reactions were purified and dried by conventional methods 24.

C, H and N analyses were performed with the Carlo-Erba LA-118 microdosimeter and the AAS-1N Carl-Zeiss-Jena spectrometer was used for the determination of Cu(II) and Ni(II). Vanadium was determined following the method described by Fries and Getrost 25. Infrared spectra (4000–400 cm^−1^) were recorded on a Bruker Vertex 70 (Billerica, Mssachusett, USA) spectrophotometer, using KBr pellets. The ^1^H NMR and ^13^C NMR spectra were recorded on a Bruker 400 DRX Billerica, Mssachusett, USA spectrometer in DMSO solution, using TMS as the internal standard. Diffuse reflectance spectra were recorded on a Jasco V-670 (Tokyo, Japan) spectrophotometer, using MgO dilution matrices. Electron paramagnetic resonance (EPR) measurements were performed on polycrystalline powders and DMSO solutions, at room -temperature and 77 K, with an MiniScope MS200; Magnettech Ltd. (Berlin, Germany) X-band spectrometer (9.3–9.6 GHz), connected to a PC equipped with a 100 kHz field modulation unit. The g factors were quoted relative to the standard marker tetracyanoethylene (g = 2.00277). Magnetic susceptibility measurements were performed at room temperature in the polycrystalline state on a Faraday magnetic balance (home-made). The molar conductance of the complexes in dimethylformamide solutions (10^−3^ M), at room temperature, were measured using a Consort type C-533 conductivity instrument.

#### X-ray crystallography

Crystallographic measurements for (**HL**), **2**, **4**, **5** were carried out with an Oxford-Diffraction XCALIBUR E CCD diffractometer equipped with graphite-monochromated Mo K_α_ radiation. The unit cell determination and data integration were carried out using the CrysAlis package of Oxford Diffraction 26. All structures were solved by direct methods using SHELXS-97 27 and refined by full-matrix least-squares on F_o_^2^ with SHELXL-97 27. All atomic displacements for non-hydrogen, non-disordered atoms were refined using an anisotropic model. All H atoms attached to carbon were introduced in idealized positions using the riding model with their isotropic displacement parameters fixed at 120% of their riding atom. Positional parameters of the H attached to N and O atoms were obtained from difference Fourier syntheses and verified by the geometric parameters of the corresponding hydrogen bonds. The structure of **2** was found to be a non-merohedral twin and it was treated as two-component system using tools of CrysAlis package. The twin data reduction module created a single hklf5 file containing reflections from both lattices, where an overlap decomposition algorithm was employed to resolve the overlapping reflections. This file was used for final structure refinement in conjunction with a BASF parameter that was equal to 0.57431. There were not practically reflections beyond of theta 24.1° because the low quality of the studied crystal, but the theta range (2.83–24.10°) for data refinement was enough to investigate the molecular *architecture of the given complex*. The coordinates of the reference atoms of all studied complexes were deposited with the Cambridge Crystallographic Data Centre.

The geometric parameters were calculated and the figures were drawn with the use of the PLATON program 26. The hydrogen atoms not involved in the hydrogen bonding were omitted from the generation of the packing diagrams. The disordered water molecule was not included in the packing diagram of **4**.

### Synthesis of the 1-phenyl-3-methyl-4-benzoyl-5-pyrazolone 4-ethylthiosemicarbazone

**Scheme 1:**
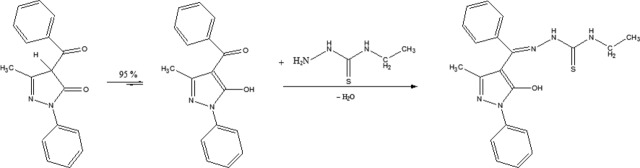
Enolisation mechanism and reaction of condensation in solution.

Previously, the two keto-enol tautomeric forms of 1-phenyl-3-methyl-4-benzoyl-5-pyrazolone (*Phmbp*) in DMSO were investigated by ^1^H NMR and ^13^C NMR spectroscopy. ^1^H NMR (DMSO- d_6_; δ, ppm): 2.28 (s-CH_3_); 4.00 wide line (OH + H_2_O from DMSO); 7.30–7.70 m (CH – benzene); ^13^C NMR (DMSO- d_6_; δ, ppm): 14.74 CH_3_; 104.79, 137.47, 157.56 (C – pyrazole); 121.70, 126.68, 128.50, 129.20, 129.49, 132.28 (CH – benzene); 139.35, 150.56(C – benzene); 190.03 C=O. The equilibrium process is shifted to the enolic form of the exocyclic carbonyl from the pyrazole ring. The enolic tautomeric form of *(Phmbp)* predominates in solution and the condensation reaction with thiosemicarbazide is shown in Scheme1.

A solution of 1-phenyl-3-methyl-4-benzoyl-5-pyrazolone (0.278 g, 1 mmol) in methanol (10 ml) was added to a solution of 4-ethylthiosemicarbazide (0.119 g, 1 mmol) in methanol (20 ml). The mixture was stirred for 2 hrs, at room temperature and refluxed at 70–80°C for 4 hrs. The resulting yellow precipitate was filtered, washed with methanol and recrystallized from methanol-ethanol (1:2, v/v). Fine yellow crystals obtained upon slow evaporation at room temperature were characterized, including the use of single crystal X-ray diffraction. Yield: 77%; M.p. 182–183°C; M.wt.: 379; *Anal*. Calc. for C_20_H_21_N_5_OS: C, 63.32; H, 5.54; N, 18.46. Found: C, 64.02; H, 4.94; N, 18.59%.

IR (KBr, cm^−1^): υ(N^4′^H) 3357, υ(N^2′^H) 3170, υ(C-O) 1230, υ(CH=N^1^) 1612, υ(N^1′^-N^2′^) 1015, υ(C=S+C=N, C=S) 1280, 849.

^1^H NMR (DMSO-d_6_; δ, ppm): 1.17 (t, J = 7.15 Hz, 3H ethyl), 1.79 (s, 3H methyl); 3.64 (q, J = 7.15 Hz, 2H ethyl); 7.30–7.98 (m, 10H benzene), 8.5 (s, 1H, NH-); 10.31 (s, 1H, NH-N=).

^13^C NMR (DMSO-d_6_; δ, ppm): 13.49 CH_3_(ethyl); 14.99 CH_3_-; 39.09 CH_2_ (ethyl); 116.74; 120.96; 124.67; 126.36; 128.26; 128.54; 128.84; 129.29; 129.53; 129.82; 130.90; 137.29; 137.68, 147.55 (benzene and pyrazole); 148.0 C=N; 177.74; C=S.

### General procedure for the preparation of the metal complexes

Complexes were prepared by a direct reaction between the ligand and the corresponding metal salts.

#### Synthesis of the complex [Cu(L)(Cl)]·C_2_H_5_OH (**1**)

A hot solution of CuCl_2_·2H_2_O (0.256 g, 1.5 mmol) in ethanol (10 ml) was added to a hot solution of HL (0.568 g, 1.5 mmol) in ethanol (15 ml). The mixture was stirred for 2 hrs at 50–60°C. The brown precipitate formed was separated by filtration, washed with cold ethanol and recrystallized from methanol-ethanol (1:1, v/v). X-ray quality single crystals were obtained.

Yield: 73%; M.wt.: 523.5; *Anal*. Calc. for C_22_H_26_CuClN_5_O_2_S: C, 50.42; H, 4.96; Cu, 12.22; Cl, 6.78; N, 13.37. Found: C, 50.83; H, 4.63; Cu, 12. 02; Cl, 6.54; N, 13.18%.

IR (KBr, cm^−1^): υ(N^4^-H) 3356; υ(N^2^-H) 3171; υ(C-O) 1214; υ(C=N) 1598; υ(C=S+C=N, C=S) 1262, 833. The complex is soluble in DMF and DMSO, and is partially soluble in ethanol and methanol.

#### Synthesis of the complex [Cu(L)_2_]·H_2_O (**2**)

The ligand HL (0.758 g, 2 mmol) dissolved in hot methanol (15 ml) and 3–4 drops of glacial acetic acid was added to Cu(OAc)_2_·H_2_O (0.200 g, 1 mmol) dissolved in ethanol (15 ml). The mixture was refluxed for 2 hrs at 50–60°C. The resulting solution was left to stand at room temperature and after several days, green-brown X-ray quality single crystals were obtained.

Yield: 75%; M.wt.: 838; *Anal*. Calc. for C_40_H_42_CuN_10_O_3_S_2_: C, 57.27; H, 5.01; Cu, 7.63; N, 16.70. Found: C, 57.45; H, 4.89; Cu, 7.51; N, 16.57%.

IR (KBr, cm^−1^): υ(N^4^-H) 3354; υ(N^2^-H) 3149; υ(C-O) 1230; υ(C=N) 1600; υ(C-S) 601. The complex is soluble in DMF and DMSO, and is partially soluble in ethanol and methanol.

#### Synthesis of the complex [Cu(L)(Br)]·H_2_O (**3**)

##### Purple solid

Yield 77%; M.wt.: 522; *Anal*. Calc. for C_20_H_20_CuBrN_5_OS: C, 45.97; H, 3.83; Cu, 12.26; Br, 15.32; N, 13.40. Found: C, 46.12; H, 3.68; Cu, 12.08; Br, 15.14; N, 13.22%.

IR (KBr, cm^−1^): υ(N^4^-H) 3354; υ(N^2^-H) 3172; υ(C-O) 1209; υ(C=N) 1597; υ(C=S+C=N, C=S) 1260, 830. The complex is soluble in DMF and DMSO and insoluble in ether.

#### Synthesis of the complex [Cu(L)(NO_3_)]·2CH_3_CH_2_OH (**4**)

##### Dark solid

Yield: 69%; M.wt.: 504; *Anal*. Calc. for C_20_H_20_CuN_6_O_4_S: C, 47.60; H, 3.96; Cu, 12.69; N, 16.66. Found: C, 47.81; H, 3.69; Cu, 12.48; N, 16.52%.

IR (KBr, cm^−1^): υ(N^4^-H) 3356; υ(N^2^-H) 3170; υ(C-O) 1211; υ(C=N) 1602; υ(C=S+C=N, C=S) 1258, 832. The complex is soluble in DMF, DMSO, partially soluble in ethanol, methanol and insoluble in ether.

#### Synthesis of the complex [VO_2_(L)]·2H_2_O (**5**)

##### Pale yellow solid

Yield: 71%; M.wt.: 497; *Anal*. Calc for C_20_H_24_VN_5_O_5_S: C, 48.28; H, 4.82; V, 10.26; N, 14.08. Found: C, 48.42; H, 4.67; V, 10.02; N, 13.92%.

IR (KBr, cm^−1^): υ(N^4^-H) 3355; υ(N^2^-H) 3168; υ(C-O) 1213; υ(C=N) 1597; υ(C=S+C=N, C=S) 1261, 831. The complex is soluble in DMF and DMSO, partially soluble in ethanol and insoluble in ether.

#### Synthesis of the complex [Ni(L)_2_]·H_2_O (**6**)

##### Green solid

Yield: 69%; M.wt.: 888; *Anal*. Calc for C_43_H_47_NiN_11_O_3_S_2_: C, 58.10; H, 5.29; Ni, 6.64; N, 17.34. Found: C, 58.32; H, 5.08; Ni, 6.48; N, 17.14%.

IR (KBr, cm^−1^): υ(N^4^-H) 3357; υ(N^2^-H) 3151; υ(C-O) 1230; υ(C=N) 1603; υ(C-S) 589. The complex is soluble in DMF, DMSO, partially soluble in ethanol, methanol and insoluble in ether.

## Results and discussion

### Antibacterial and antifungal activity

Experimental results obtained from the study of antimicrobial activity (Table1) demonstrate that the ligand (**HL**) is not active but the copper complexes **1**, **2**, **3**, **4** have bacteriostatic and bactericidal activity in a concentration range of 1.5–30 μg/ml towards both Gram-positive and Gram-negative bacteria. In comparison, the antimicrobial data characteristics for *furacillinum* used in medical practice are given. The antimicrobial activity displayed by the copper complexes **1**, **2**, **3**, **4** is 3–6 times higher towards Staphylococcus aureus and Bacillus cereus than *furacillinum* and exceeds by 22–25 times the bacteriostatic activity towards the majority of Salmonella abony Gram-negative bacteria. The MIC and MBC are influenced by the presence of copper in the composition of coordination compounds.

**Table 1:** Antibacterial and antifungal activities of ligand (HL) and complexes 1–4 as MIC/MBC values (μg/ml)

Compounds	*E. coli* (G-)		*S. abony* (G-)		*S. aureus* (G+)		*B. cereus* (G+)		*C. albicans*	
	MIC	MBC	MIC	MBC	MIC	MBC	MIC	MBC	MIC	MBC
**HL**	>600	>600	>600	>600	>600	>600	>600	>600	>600	>600
[Cu(L)(Cl)] C_2_H_5_OH (**1)**	>600	>600	3.0	7.0	15	30	3.0	15	1.5	3.0
[Cu(L)_2_]·H_2_O (**2**)	>600	>600	1.5	7.0	7.0	1.5	1.5	3	1.5	1.5
[Cu(L)(Br)] H_2_O (**3**)	>600	>600	3.0	7.0	3.0	70	1.5	3	1.5	1.5
[Cu(L)(NO_3_)]·2CH_3_CH_2_OH (**4**)	>600	>600	4.0	6.0	4.0	6.0	1.2	1.2	1.4	4.0
CuCl_2_·2H_2_O	>600	>600	>600	>600	>600	>600	>600	>600	>600	>600
CuBr_2_	>600	>600	>600	>600	>600	>600	>600	>600	>600	>600
Cu(NO_3_)_2_·3H_2_O	>600	>600	>600	>600	>600	>600	>600	>600	>600	>600
Furacillinum	18.5	37.5	75	150	9.35	9.35	9.35	18.5	–	–
Nystatine	–	–	–	–	–	–	–	–	80	80

*E. coli* (*Escherichia coli,* ATCC 25922); *S. abony* (*Salmonella abony,* NCTC 03/03); *S. aureus* (*Staphylococcus aureus*, ATCC 25923); *B. cereus* (*Bacillus cereus*, NCTC 8035); *C. albicans* (*Candida albicans*); MIC, minimum inhibitory concentration; MBC, minimum bactericide concentration.

The data concerning the study of antimycotic properties of the copper complexes **1**, **2**, **3**, **4** show that they also display bacteriostatic and bactericidal activity in a concentration range of 1.5–4.0 μg/ml towards Candida fungi. For comparison, we also added data regarding the activity of nystatine, a compound used in medicine for mycoses treatment. The results show that the copper complexes have antimycotic activity against Candida fungi, 20–25 times higher than that of nystatine.

The antibacterial results evidently show that the activity of the Schiff base compounds became more pronounced when coordinated to the metal ions. This is probably due to the greater lipophilic nature of the complexes. Such increased activity of the metal chelates can be explained on the basis of chelating theory 28. Upon chelation, the polarity of the metal ion is reduced to a greater extent as a result of the overlap of the ligand orbital and partial sharing of the positive charge of the metal ion with donor groups. Further, it increases the delocalization of π-electrons over the whole chelate ring and enhances the lipophilicity of the complex. This increased lipophilicity enhances the penetration of the complexes into lipid membrane and blocks the metal binding sites on enzymes of micro-organisms.

### Antileukaemia activity

The ligand (1-phenyl-3-methyl-4-benzoyl-5-pyrazolone 4-ethyl-thiosemicarbazone) and its metal complexes (Table2) were tested as inhibitors of HL-60 cell proliferation using three concentrations: 0.1, 1.0 and 10 μmol/L. At all concentrations the ligand did not have inhibitory activity. Therefore, the presence of the metal ion in the Schiff bases composition is important. This fact is confirmed specially for copper complexes. The copper complexes, including the tridentate ONS ligand, demonstrate an important antiproliferative activity for HL-60 leukaemia cells compared to those containing inner sphere halogen.

**Table 2:** Antiproliferative activity of ligand and metal complexes on human leukaemia HL-60 cells at three concentrations

Compound	Inhibition of cell proliferation (%)*		
	10 μM	1 μM	0.1 μM
**HL**	0	0	0
[Cu(L)(Cl)]·C_2_H_5_OH (**1)**	98.9	41.3	2.0
[Cu(L)_2_]·H_2_O (**2**)	99.9	96.0	5.0
[Cu(L)(Br)]·H_2_O (**3**)	98.8	35.5	0
[Cu(L)(NO_3_)]·2CH_3_CH_2_OH (**4**)	96.8	45.8	4.0
[VO_2_ (L)]·2H_2_O (**5**)	4.0	0	0
[Ni(L)_2_]·H_2_O (**6**)	5.7	0	0
CuCl_2_·2H_2_O	0	0	0
(VO)SO_4_·2H_2_O	0	0	0
NiCl_2_·6H_2_O	0	0	0

* SEM < ±4% of a single experiment in triplicate.

If copper is capsulated with two ligands (complex **2**), the antiproliferative activity is the highest and the concentration dependence changes from 5.0% to 99.9%. The antiproliferative activity of the copper complexes is of the same significance as for doxorubicin, which is utilized in medicinal practice as antileukemia drug.

Finally, the nature of metal ion in the coordination compound is very important and the antiproliferative activity dramatically decreases for vanadium and nickel.

The physico-chemical analyses confirmed the composition and the structure of the newly obtained complex combinations. Depending on the metal salt anion used, the ligand acts as a mononegative tridentate (**1**, **3**, **4**, **5**) through the thioenolic sulphur, the azomethine nitrogen and exocyclic carbonyl oxygen of the pyrazol moiety or a mononegative bidentate through the thioenolic sulphur and the azomethine nitrogen (**2**, **6**). The sensitivity spectrum of the microbial strains towards the ligand and the corresponding complexes was determined by qualitative and quantitative methods.

The antimicrobial data given for the compounds presented in this article showed that the metal complexes generally have better activity than the free ligand. The antimicrobial activity was dependent on the microbial species tested and metal salt anion used.

The antiproliferative activity towards HL-60 cells of some complex combinations occurred in a concentration-dependent manner in the range of 1–10 μM, while a similar effect, was not exhibited by the ligands alone or the copper salts used for the metal complex synthesis at identical tested concentrations. The complex **2**, which is capsulated with two ligands, demonstrates the highest antiproliferative activity of the series of compounds.

### Chemistry

New metal compounds **1**–**6** were synthesized in methanol with good yield. They are microcrystalline solids that, decomposed above 250°C, are soluble in organic solvents such as DMF, DMSO, chloroform, but insoluble in ether. The molar conductance values of the soluble complexes in DMF (8–15 ohm^−1^ cm^2^/mol) showed that complexes **1**–**6** are non-electrolytes 29. The elemental analyses data of the thiosemicarbazone and its complexes (see Experimental) were compatible with the structures of the ligand and of the complexes.

The green and brown colours are common to complexes involving thiosemicarbazone coordination because of the sulphur-to-metal charge-transfer bands, which dominate their visible spectra 30.

### Structural characterization of the ligand (HL) and the complexes (2, 4 and 5)

**Fig 1:**
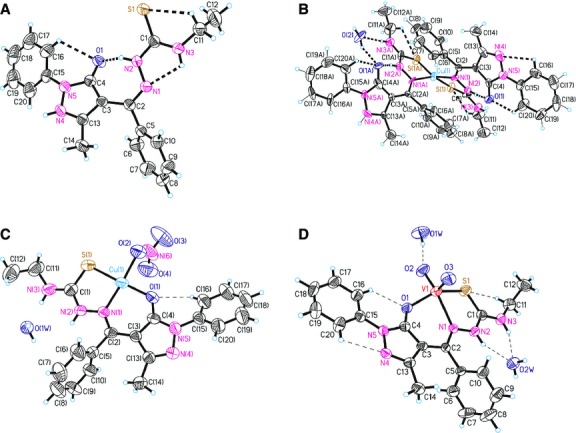
ORTEP drawing for compounds HL, 2, 4, 5 with the atomic labelling. Thermal ellipsoids are shown with the 50% probability level.

**Fig 2:**
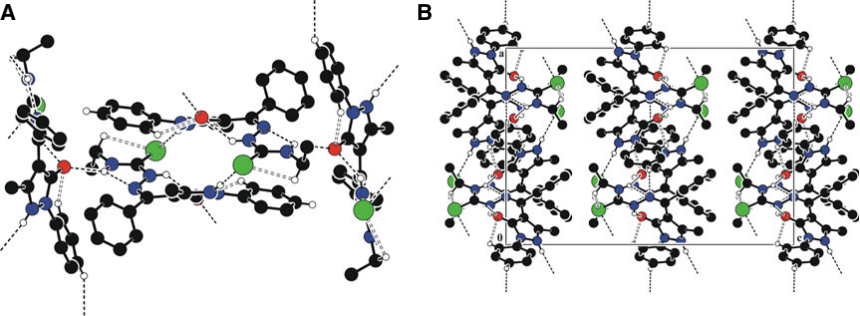
(A) Crystal packing of HL representing the *2-D* layers parallel to (*001*) plane. (B) Fragment of molecular packing in the crystal of HL.

**Fig 3:**
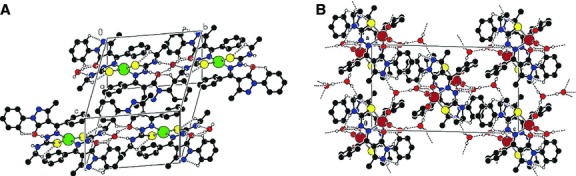
(A)The crystal packing of 2 showing the formation of chains which are aligned along [0 1 0] direction because of water molecules. (B) The crystal packing showing the *3D* hydrogen-bonded network built from complexes of 5.

**Fig 4:**
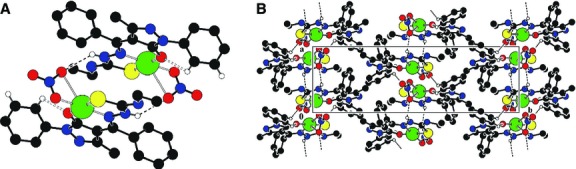
(A) The dimer formation where the complexes are linked by NO_3_-groups. (B) The crystal packing of 4 representing the consolidation of dimers into chains aligned along [1 0 0] direction.

Crystals of (**HL**), [Cu(L)_2_]·H_2_O (**2**), [Cu(L)(NO_3_)]·2CH_3_CH_2_OH (**4**), [VO_2_(L)]·2H_2_O (**5**), suitable for single crystal X-ray study were grown from acetonitrile, by slow evaporation. The principal crystallographic data and the refinement details are summarized in Table3. Selected bond lengths and angles are presented in Table4. The single crystal X-ray study revealed that all the compounds have a molecular structure built from the neutral entities depicted in Figures1–4.

**Table 3:** Crystallographic data, details of data collection and structure refinement parameters for compound (HL), 2, 4 and 5

Compound	HL	2	4	5
Chemical formula	C_20_H_21_N_5_OS	C_40_H_42_ CuN_10_O_3_S_2_	C_20_H_20_CuN_6_O_4_S	C_20_H_24_VN_5_O_5_S
M (g/mol)	379.47	838.5	508.02	497.44
Temperature (K)	293	293	293	293
Wavelength (Å)	0.71073	0.71073	0.71073	0.71073
Crystal system	Orthorhombic	Triclinic	Monoclinic	Monoclinic
Space group	*Pbcn*	*P-1*	*P2*_*1*_*/c*	*P2*_*1*_*/n*
a (Å)	14.5376(6)	11.374(2)	13.534(5)	10.9731(6)
b (Å)	12.7713(4)	12.375(2)	23.495(2)	10.6930(7)
c (Å)	21.1856(7)	14.829(2)	7.5128(5)	19.0149(13)
α (^0^)	90	99.935(14)	90	90
β (^0^)	90	98.607(13)	96.163(10)	91.424(6)
γ (^0^)	90	97.504(15)	90	90
V (Å^3^)	3933.4(3)	2006.3(6)	2375.2(5)	2230.4(2)
D_calc_ (g/cm^3^)	1.282	1.388	1.421	1.481
μ (mm^−1^)	0.184	0.700	1.046	0.581
F(0 0 0)	1600	874	1044	1032
Goodness-of-fit on F^2^	0.982	0.600	1.047	0.971
Final R_1_, wR_2_ [I > 2σ(I)]	0.0497	0.0575	0.0691	0.0683
	0.1045	0.0597	0.1783	0.0937
R_1_, wR_2_ (all data)	0.0891	0.2785	0.1142	0.1420
	0.1178	0.0927	0.2003	0.1130
Largest difference in peak and hole (e Å^−3^)	0.256, −0.222	0.257, −0.263	0.744, −0.362	0.509, −0.348

**Table 4:** Selected bond lengths and angles for (HL), 2, 4 and 5

Bond angels	ω, deg.			
	HL	2	4	5
Cu(1) [V(1)]-S(1)		2.246(2)	2.2471(16)	2.3647(15)
Cu(1) [V(1)]-S(1A) [O(1)]		2.258(3)	1.916(4)	1.904(3)
Cu(1) [V(1)]-N(1)		1.987(5)	1.965(4)	2.244(3)
Cu(1) [V(1)]-N(1A) [O(2)]		1.973(6)	2.001(5)	1.638(3)
S(1)-C(1) (S(1A)-C(1A))	1.695(2)	1.693(8) (1.706(8)	1.697(6)	1.696(4)
N(1)-N(2)	1.374(2)	1.376(7)	1.363(6)	1.390(4)
N(1)-C(2)	1.293(3)	1.307(7)	1.325(6)	1.313(5)
N(2)-C(1)	1.359(3)	1.340(7)	1.349(7)	1.341(5)
N(3)-C(1)	1.313(3)	1.336(9)	1.309(7)	1.314(5)
N(3)-C(11)	1.453(3)	1.445(8)	1.496(8)	1.458(5)
C(3)-C(2)	1.470(3)	1.448(9)	1.409(7)	1.418(5)
C(3)-C(4)	1.434(3)	1.436(11)	1.402(7)	1.399(5)
C(4)-N(5)	1.384(3)	1.396(9)	1.330(7)	1.360(5)
O(1)-C(4)	1.250(3)	1.244(8)	1.279(6)	1.300(4)
N(5)-N(4)	1.384(3)	1.370(8)	1.404(6)	1.384(4)
C(13)-N(4)	1.331(3)	1.309(10)	1.298(6)	1.326(5)
N(5)-C(15)	1.420(3)	1.413(10)	1.432(6)	1.419(5)
C(13)-C(14)	1.494(3)	1.519(10)	1.490(7)	1.496(5)
**Bond angles**	**ω, deg.**			
N(1A) [O(2)]-Cu(1) [V(1)]-N(1)		105.3(2)	175.1(2)	153.26(15)
N(1A) [O(2)]-Cu(1) [V(1)]-S(1)		148.0(2)	89.17(15)	86.58(12)
N(1)-Cu(1) [V(1)]-S(1)		85.42(19)	87.22(13)	78.23(9)
N(1A) [O(2)]-Cu(1) [V(1)]-S(1A)[O(1)]		86.2(2)	89.28(18)	95.64(14)
N(1)-Cu(1) [V(1)]-S(1A) [O(1)]		147.5(2)	94.39(16)	83.26(12)
S(1)-Cu(1) [V(1)]-S(1A) [O(1)]		100.88(9)	178.14(11)	141.32(10)
C(1)-S(1)-Cu(1) [V(1)]		95.1(3)	96.5(2)	102.61(17)
N(3)-C(1)-S(1)	124.58(18)	122.8(6)	122.9(4)	123.1(3)
N(2)-C(1)-S(1)	117.91(17)	121.4(6)	119.6(4)	119.9(3)
N(3)-C(1)-N(2)	117.4(2)	115.5(7)	117.4(5)	117.0(4)
N(1)-C(2)-C(3)	127.5(2)	127.8(8)	120.1(5)	118.5(4)
O(1)-C(4)-N(5)	123.3(2)	125.4(9)	121.9(5)	123.4(4)
O(1)-C(4)-C(3)	130.7(2)	132.1(8)	130.8(5)	128.4(4)
N(4)-N(5)-C(4)	108.51(19)	113.6(8)	111.7(4)	110.4(3)
N(4)-C(13)-C(3)	109.9(2)	112.5(9)	111.4(4)	111.7(4)
N(4)-C(13)-C(14)	118.0(2)	117.2(8)	118.1(5)	118.0(4)

In (**HL**), the substituents at the N(2)–C(1) bonds are in the *E* positions. The S(1)-N(1)-N(2)-N(3)-C(1)-C(2) core of the ligand essentially planar to within 0.075 Å but the molecule is non-planar. The best planes of the phenol (C(5)–C(10)), (*C*(3)C(4)C(13)N(4)N(5)) and (C(15)-C(20)) rings are inclined to this core at angles of 42.2°, 34.7° and 14.6°, respectively.

In complex **2** the central ion is a tetrahedral coordination environment provided by two monodeprotonated bidentate ligands, which exhibit typical (N_2_S_2_) co-ordinating behaviour for thiosemicarbazone moieties 31,32 resulting into the formation of the five-membered chelate ring. The distances between the centres of S(1) and C(1) atoms, for complexes **2**, **4** and **5** are 1.693(8), 1.697(6), 1.696(4) Å, respectively, which is close to the C-S single bond and from which we can deduce that the structures of the complexes have a thiol form.

In complex **4**, the copper atom is tetracoordinated with a square planar geometry while in complex **5**, the coordination polyhedron of the central ion can be described as a distorted square pyramid. In these complexes, the thiosemicarbazone ligand coordinates to the metal atom in a tridentate manner using its azomethine nitrogen, thiolate sulphur and the exocyclic carbonyl oxygen of the pyrazol moiety, resulting a five-membered chelate ring and the other six membered. The basal plane of the square pyramid of the metal atom in **5** is formed by the S1, O1, O2 and N1 atoms of the monodeprotonated (**HL**) ligands. The apexes of the metals' coordination pyramids in **5** are occupied by oxygen atoms O3 with distances of 1.605(1) Å. In complexes **4** and **5,** the distance between the centres of O(1) and C(4) atoms is 1.279(6) and 1.300(4) Å, respectively, which is similar to the value for a C=O double bond. In the crystal structure of **4**, the complexes form dimers which are linked by NO_3_^−^ groups. The dimers are joined into chains through the hydrogen bonds N2-H…O4 (1 − *x*,−*y*,1 − *z*) while in the crystal structure of **5,** the complexes are linked together by H-bonds to form a three dimensional framework. Table5 shows the H-bonding interactions of (**HL**) and complexes **2**, **4** and **5**.

**Table 5:** Hydrogen bonds in X-ray structures for (HL), 2, 4 and 5

D-H…A	d(D…H), Å	d(H…A), Å	d(D…A), Å	∠(DHA), deg.	Symmetry transformation for H-acceptor
(**HL**)					
N4-H…S1	0.86	2.38	3.2261	171	1 − *x*,1 − *y*,−*z*
N2-H…O1	0.86	2.02	2.7315	139	*x*,*y*,*z*
N3-H…N1	0.86	2.24	2.6225	107	*x*,*y*,*z*
N3-H…O1	0.86	2.34	3.0114	135	1/2 − *x*,−1/2 + *y*,*z*
C11-H…S1	0.97	2.79	3.1035	1100	*x*,*y*,*z*
C16-H…O1	0.93	2.46	2.9569	114	*x*,*y*,*z*
C18-H…N1	0.93	2.59	3.4532	154	1/2 + *x*,3/2 − *y*,−*z*
**2**					
N2-H…O1	0.86	1.75	2.575(7)	160	*x*,*y*,*z*
N2A-H…O1A	0.86	1.74	2.560(8)	159	*x*,*y*,*z*
N3-H…O2	0.86	2.08	2.922(10)	167	*x*,−1 + *y*,*z*
O2-H…O1	0.83(6)	1.95	2.760(9)	164	*x*,1 + *y*,*z*
N3A-H…O2	0.86	2.19	3.047(10)	175	*x*,*y*,*z*
O2-H…O1A	0.83(8)	1.95(8)	2.705(9)	151(9)	*x*,*y*,*z*
C16-H…N4	0.93	2.44	2.769(12)	101	*x*,*y*,*z*
C20A-H…O1A	0.93	2.40	2.934(11)	117	*x*,*y*,*z*
C11A-H…S1A	0.97	2.72	3.121(10)	106	*x*,*y*,*z*
C20-H…O1	0.93	2.31	2.923(10)	123	*x*,*y*,*z*
C11-H…S1	0.97	2.66	3.097(10)	108	*x*,*y*,*z*
**4**					
N2-H…O4	0.86	2.32	3.0173	139	1 − *x*,−*y*,1 − *z*
C16-H…O1	0.93	2.44	2.9235	113	*x*,*y*,*z*
C17-H…O3	0.93	2.51	3.2717	139	−*x*,−*y*,−*z*
**5**					
N3-H…O2W	0.86	2.00	2.7948	153	1 − *x*,1 − *y*,−*z*
O2W-H…O3	0.85	1.98	2.8033	164	−1 + *x*,*y*,*z*
O2W-H…O1W	0.85	1.91	2.7588	172	1/2 − *x*,1/2 + *y*,1/2 − *z*
N2-H…O2W	0.86	2.06	2.8274	148	1 − *x*,1 − *y*,−*z*
O1W-H…O2	0.85	1.89	2.7355	172	−1 + *x*,*y*,*z*
O1W-H…N4	0.85	2.22	3.0383	161	*x*,*y*,*z*
C20-H…N4	0.93	2.44	2.7778	102	*x*,*y*,*z*
C11-H…S1	0.97	2.74	3.0678	101	*x*,*y*,*z*
C16-H…O1	0.93	2.21	2.8616	126	*x*,*y*,*z*

### Infrared spectra and coordination mode

The tentative assignments of the significant IR spectral bands of (**HL**) and its complexes are reported in the Experimental section. The IR spectra of the complexes are compared with those for the free ligand to determine the changes that might occur during complex formation. The proposed assignments are based on previous results 19–21,33,34 and pertinent references 35–41.

The υ(C=N) band of the thiosemicarbazone at 1612 cm^−1^ shifted to lower frequencies (1597–1603 cm^−1^) in the complexes indicating coordination *via* the azomethine nitrogen. The υ(N-N) strong band of the thiosemicarbazone is found at 1015 cm^−1^. The increase in the frequency of this band in the spectra of the complexes is due to the increase in the double bond character (the bond strength). This is a confirmation of the coordination of the ligand through the azomethine nitrogen atom 42.

In the free ligand spectrum, the two bands appearing at the frequencies at 1280 and 849 cm^−1^ have been assigned to υ(C=S+C=N) and υ(C=S). Evidence of the coordination of the thionic sulphur is further supported by the shifting of these bands in the spectra of the complexes **1**, **3**, **4** and **5**, to a lower wave number 43–46. The shift of the υ(N^2^-H) band in the spectra of the complexes **2** and **6** indicates the deprotonation of the hydrazinic proton which is in accordance with the coordination of the sulphur atom in the thiolate form 44–46.

The band from 1230 cm^−1^ because of υ(C-O), for the exocyclic enolic group in the free ligand spectrum is shifted by 18–22 cm^−1^ to lower energies in the spectra of the complexes **1**, **3**, **4** and **5**. This indicates that the exocyclic enolic oxygen is bonded to the metallic ions 35,36,47,48. In the IR spectra for complexes **2** and **6**, υ(C-O) remains un-modified, indicating that the exocyclic ketonic oxygen of the antipyrine ring is not involved in the coordination 49.

### Electronic spectra and magnetic studies

The tentative assignments of the significant electronic spectral bands of ligand and their complexes are presented in Table6. The electronic spectrum in the polycrystalline state of ligand (**HL**) showed the intraligand absorption maxima: 38,400 and 28,900 cm^−1^, assigned to π→π* and n→π* transitions, corresponding to azomethine and thioamide groups of the ligand 50. In the spectra of the complexes, these bands are shifted to lower energies and new bands appeared in the regions 37,000–36,300 cm^−1^ and 27,700–26,300 cm^−1^.

**Table 6:** Electronic spectra (cm^−1^) and magnetic moment (BM) of the complexes 1–6

Metal complex molecular formula	Transitions d-d (cm^−1^)			μ_eff_ (BM)	Geometry
[Cu(L)(Cl)]·C_2_H_5_OH (**1**)	^2^B_1g_→^2^B_2g_ 11,200	^2^B_1g_→^2^E_g_ 15,870	^2^B_1g_→^2^A_1g_ –	1.54	Square-planar
[Cu(L)_2_]·H_2_O (**2**)	^2^B_2_→^2^E 10,500	^2^B_2_→^2^B_1_(^2^A_1_) 14,390	–	1.75	Pseudo-tetrahedral
[Cu(L)(Br)]·H_2_O (**3**)	^2^B_1g_→^2^B_2g_ 12,050	^2^B_1g_→^2^E_g_ 16,260	^2^B_1g_→^2^A_1g_ 19,250	1.69	Square-planar
[Cu(L)(NO_3_)]·2CH_3_CH_2_OH (**4**)	^2^B_1g_→^2^B_2g_ 11,900	^2^B_1g_→^2^B_2g_ 15,620	^2^B_1g_→^2^E_g_ –	1.82	Square-planar
[VO_2_(L)]·2H_2_O (**5**)			25,680(CT)	Diamagnetic	Square-pyramidal
[Ni(L)_2_]·H_2_O (**6**)	^3^A_2_→^3^T_1_(F) 10,750	^3^A_2_→^3^T_1_(P) 16,950	–	3.56	Square-planar

The spectra of the complexes **1**, **3** and **4** exhibit d–d bands corresponding to the transitions specific to the square-planar complexes with 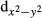 (^2^B_1g_) ground state 51. The room temperature magnetic moment values (1.50, 1.62 and 1.82 B.M) of the solid copper (II) complexes **1**, **3** and **4**, respectively, are indicative of anti-ferromagnetic interaction through molecular association for square-planar geometry 52.

In the electronic spectrum of complex **2,** a broad band was observed at 14,390 cm^−1^ due to a d_*xy*_ (^2^B_2_)→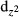 (^2^A_1_), 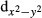 (^2^B_1_) transition, suggesting a pseudo-tetrahedral configuration around the central metal ion 51,53. A second transition appeared as a weak shoulder on the broad band. The magnetic moment of complex (1.75 B.M) is normal for copper (II) with pseudo-tetrahedral stereochemistry 52,54.

The electronic spectrum of complex **5** shows an intense and broad band, specific for complexes with metallic ion d^0^ 55.

For complex **6**, the electronic spectrum showed a two band association for tetrahedral geometry 51. The magnetic moment value (3.56 B.M) corresponding to two unpaired electrons per nickel (II) centres for square-planar configuration 52,54.

### EPR spectra

The EPR spectral parameters of the copper(II) complexes 1–4 in the polycrystalline state at 298 K and in DMSO solution at 77 K are presented in Table7. The EPR spectra of the complexes recorded in the polycrystalline and solution state provide information about the coordination environment around copper(II).

**Table 7:** EPR spectral parameters of the copper(II) complexes 1–4

	1	2	3	4
Polycrystalline (298 K)				
g_//_	2.22	2.265	2.21	2.18
g_⊥_	2.042	2.053	2.037	2.047
DMSO (77 K)				
g_//_	2.219	2.188	2.243	2.241
g_⊥_	2.051	2.077	2.062	2.062
A_//_	177	174	173	175
α^2^	0.7691	0.7410	0.7867	0.7903
β^2^	0.9366	0.8570	0.9752	0.9491
δ^2^	0.7460	0.9286	0.8376	0.8286
K_//_	0.7204	0.6351	0.7672	0.7501
K_⊥_	0.5738	0.6881	0.6590	0.6549

**Fig 5:**
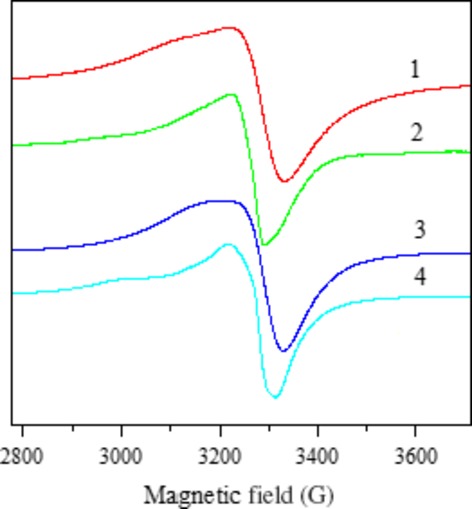
EPR spectra of 1-4 in the polycristalline state at the room temperature.

The spectra of the compounds 1–4 in the polycrystalline state show only one broad signal, and typical axial behaviour with slightly different g_//_ and g_⊥_ values (Fig.5). In these complexes, tensor values of g_//_ > g_⊥_ > 2.0023 are consistent with a 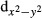 ground state 56.

**Fig 6:**
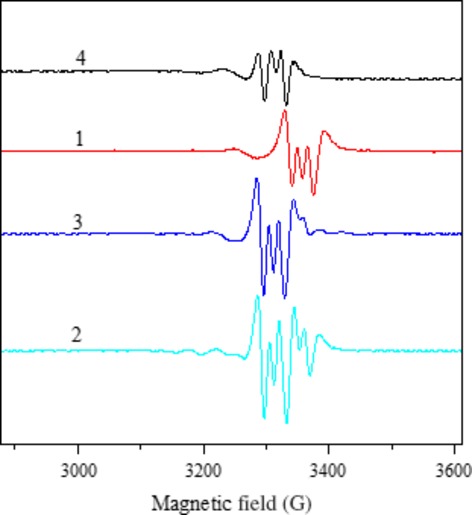
EPR spectra of 1-4 in DMSO solution at the room temperature (second derivative).

The solution spectra of the complexes were recorded in DMSO at 298 K. The spectra of the complexes **1**, **3** and **4** show three nitrogen superhyperfine lines and five for complex **2**, in the high field component (Fig.6). The g_//_/A_//_ratio can be used as a empirical convenient of distortion from square-planar structure 57,58. The values of the g_//_/A_//_ratio for complexes **1**, **3** and **4** indicate nearly square-planar environments with small distortions, which is in good agreement with the X-ray determined structure for complex **4**.

**Fig 7:**
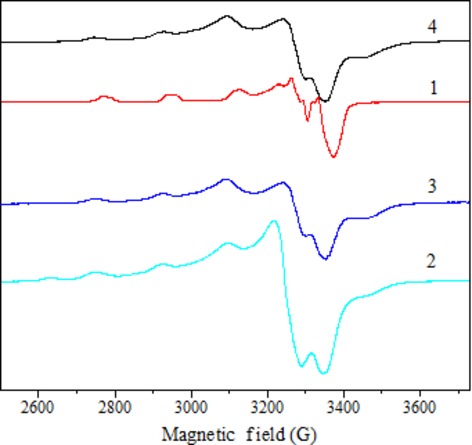
EPR spectra of 1-4 in DMSO solution at 77 K.

The spectra of all the complexes in frozen DMSO at 77 K are axial with four copper hyperfine lines in the parallel region (Fig.7). Additionally, in all these complexes, g_//_ > g_⊥_ > 2.0023 corresponding to the presence of an unpaired electron in the 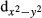 orbital 56. The energies of d–d transition and the EPR spectral parameters g_//_, g_⊥_ and A_//_, were used to evaluate the bonding parameters α^2^, β^2^, δ^2^. These parameters may be regarded as measures of the covalency of the in-plane σ bonds, in-plane p bonds and out-of-plane π bonds 40,59.

The orbital reduction factors K_//_ and K_⊥_ were calculated using expressions reported elsewhere 40,60,61. The K_//_ and K_⊥_ values, in complexes **1**, **3** and **4**, are in agreement with the relation K_//_ > K_⊥_. The values of these factors indicate the presence out-of-plane π bonding. For complex **2**, these values indicate the presence of in-plane π bonding (K_//_ < K_⊥_).

## Appendix A: Supplementary data

CCDC 930999, 950024, 931000 and 931001 contain the supplementary crystallographic data for C_20_H_21_N_5_OS (**HL**), C_40_H_42_CuN_10_O_3_S_2_ (**2**), C_20_H_20_CuN_6_O_4_S (**4**) and C_20_H_24_VN_5_O_5_S (**5**). These data can be obtained free of charge *via* http//www.ccdc,cam.ac.uk/conts/retrieving.html, or from the Cambridge Crystallographic Data Centre, 12 Union Road, Cambridge CB2 1EZ, UK; fax: (+44) 1223-336-033; or e-mail: deposit@ccdc.cam.ac.uk.
